# Violence against Children: A Challenge for Public Health in Pakistan

**Published:** 2007-06

**Authors:** Adnan Ali Hyder, Fauzia Aman Malik

**Affiliations:** Department of International Health, Bloomberg School of Public Health, Johns HopkinsUniversity, 615 N. Wolfe Street, Suite E8142, Baltimore, MD 21205, USA

**Keywords:** Child abuse, Human development, Public health, Violence, Pakistan

## Abstract

World Health Organization has identified violence against children as a growing public-health issue with a global magnitude. This paper explored violence against children as a challenge in the developing world using Pakistan as a case study. A systematic review of existing research and literature on violence against children was followed by assessing the magnitude of this challenge and its impact on policy. Most research done in Pakistan is observational, descriptive, and anecdotal with data collected through survey methods and interviews with small sample sizes. The findings suggest that the confluence of macro risk factors, such as poverty, poor legal protections, illiteracy, large family size, and unemployment, create an enabling environment for violence against children. Lack of empirical data makes it difficult to assess the magnitude of this issue. The health problems reported and the extent of human potential destroyed are unknown. Conclusion calls for focused research to examine the prevalence, potential interventions, and policies in Pakistan.

## INTRODUCTION

The World Report on Violence and Health, released by the World Health Organization (WHO) (1), presented the issue of violence against children as a public-health problem that has a global magnitude. In the same year (2002), the member nations of the United Nations (UN) pledged to meet eight Millennium Development Goals by 2015 (2). Six of these goals are directly related to children, and all are closely linked to the commitment made at the Special Session on Children of the UN General Assembly in 2002 that all governments would work to promote and protect the rights of every child (3).

WHO defines violence as “The intentional use of physical force or power, threatened or actual, against oneself, another person, or against a group or community that either results in or has a high likelihood of resulting in injury, death, psychological harm, maldevelopment or deprivation”(1). This definition captures the range of potential and actual violence perpetuated on people, including children, the most vulnerable group. For example, an estimated 57,000 deaths have been attributed to homicide among children aged less than 15 years in 2000 (3). The global estimates of child homicide suggest that infants and very young children, aged 0-4 year(s), are at the highest risk, while children in lower-income countries are at a higher risk compared to those in high-income countries. The highest rates of homicide for children aged less than five years are in the African Region (AFRO) at 17.9 per 100,000 for boys and 12.7 per 100,000 for girls (3).

Violence against children occurs in different forms (physical, sexual, neglect, emotional and psychological) and at multiple levels (individual, household, institutional, and societal). A WHO Consultation on Child Abuse Prevention recognized violence against children as a growing public-health and development problem and defined child abuse as “Child abuse and maltreatment constitutes all forms of physical and/or emotional ill-treatment, sexual abuse, neglect or negligent treatment or commercial or other exploitation, resulting in actual or potential harm to the child's health, survival, development or dignity in the context of a relationship of responsibility, trust or power” (3). Establishing the precise magnitude of child abuse for any given country is very difficult. Even in wealthy countries, recognizing and measuring the incidence of fatal violence such as infanticide is problematic due to underreporting and misclassification of deaths. The situation in developing countries is even more challenging due to a mix of poor health-information systems, faulty legal and police structures, and sociocultural stigma (3). Data on non-fatal abuse is even harder to collect because of different legal and cultural definitions of abuse and neglect across countries. Such cases are more underreported even in countries where mandatory reporting mechanisms exist.

Violence against children occurs throughout the world, including South Asia, which has 23% of the world's population and is one of the world's poorest regions (4). In countries, like India, Bangladesh, Bhutan, Nepal, and Pakistan, child labour, child sexual abuse and prostitution, child trafficking and homelessness are commonly reported issues. The largest number of working children in the world—between 40 and 115 million child workers aged 5-14 years—are found in India (5). Data from a 1995 Bangladesh survey of children aged 10-15 years in 150 villages revealed that 21% were in the labour force (6).

Pakistan is a developing country in South Asia with a population of 153 million and a per-capita gross national income of US$ 420 (4). Of the total population, 43% are children aged less than 15 years (7), and according to the World Bank (4), poverty remains a serious concern in Pakistan, with 33% of the population living under the poverty-line, with a literacy rate of 44%. Pakistan ranks 138 on the Human Development Index (8). Pakistan is struggling to make its general and specific environment conducive for meaningful and sustainable advancement in terms of all aspects of human development. Importantly, Pakistan ratified the UN Convention on the Rights of the Child in 1990.

The paper examines the situation of violence against children in Pakistan. Its overall goal is to assess the current state of knowledge on violence against children in the country. The specific objectives are to conduct a systematic review of the literature on violence against children, to understand the nature and context of this problem, to estimate the magnitude of violence against children in Pakistan as a public-health problem, and to define critical gaps in existing knowledge for public-health research and policy.

## MATERIALS AND METHODS

A comprehensive review of literature on violence against children in Pakistan was undertaken. In addition to published articles that contained qualitative and quantitative data from original research or review, organizational reports and unpublished items were also reviewed. PubMed was searched for literature published in English from 1966 to 2003. Combinations of key words, such as ‘violence against children’, ‘abuse’, ‘child maltreatment’, ‘pediatric violence’, ‘neglect’, ‘child labor’, ‘child sexual abuse’, ‘child trauma’, ‘Pakistan’, and ‘South Asia’, were used. Further searches were conducted using the author's name and ‘related articles’ links for key publications. A separate search was conducted in www.pakmedinet.com (electronic database) to identify literature from local health journals within Pakistan. A Web-based www.google.com search was also done to retrieve government publications and institutional reports released by international, non-governmental, academic organizations, and research centres. Organizations, such as United Nations Children's Fund (UNICEF), Human Rights Commission of Pakistan, WHO, and International Labour Organization (ILO), were searched using the same key words as above.

Abstracts of each paper of potential interest were reviewed by both the authors for inclusion in the study, and full copies of these publications were obtained. Literature was included in the review if it was based on a population living within the geographical boundaries of Pakistan, in English, relating to any type of violence against children or included information on children aged less than 18 years. Articles were excluded if participant populations were outside Pakistan, represented violence against adults aged over 18 years only, or if they were news reports. Each paper was reviewed to extract qualitative and quantitative data on violence against children. These were tabulated and summarized to assess the magnitude and characteristics of violence against children in the country. A quantitative analysis was done to see if rates of prevalence and summary distributions could be established. Qualitative analysis explored the type, design, and location of each study and evaluated recurring themes and relations for causes of violence.

## RESULTS

The systematic review of literature on violence against children in Pakistan identified 15 published papers—three editorials, 10 original research papers, and two review papers—written between 1984 and 2003 (Table 1). Unpublished literature covered 11 organizational reports generated during 1998-2003 (Table 2). The following main themes predominated the literature on violence against children: child labour (9–18); sexual abuse (16,19,20–23); child neglect, as it manifests in the form of lack of education, gender discrimination, and malnutrition, particularly for the girl child (19,20,22,24–26); juvenile law (16–19); and impact of political or street violence on children (16,19,26,27). Most published studies were from national medical journals, had an urban bias towards larger cities of Pakistan, and the populations studied were small, except for one study from Karachi city that screened 112,029 children (28). The appearance of empirical work in the published literature reflected some research on violence against children in the country, while reviews and situation analysis dominated the unpublished literature.

**Table 1:** Violence against children in Pakistan: peer-reviewed published papers

Source	Setting	Population studied	Type of study/ methods	Primary topic	Secondary themes
Miller 1984	Pakistan, Bangladesh, and India/ predominantly rural (some urban)	Boys and girls aged less than 10 years	Cross-cultural study/ethnography/ based on previous studies, national data	Daughter neglect	Juvenile sex ratios, mortality, women's work roles and marriage patterns
Aftab 1991	Lahore (urban area)	360 working boys and girls (10 occupational groups)	Arbitrary sampling, interview survey	Child labour	Poverty, illiteracy, survival, rural-urban migration, labour laws, and juvenile delinquency
Talaat 1996	Urban Peshawar	30 working boys aged 9-17 years (83.7% 12-17 years)	Interviews and observations	Child labour in low-class hotels and restaurants	Social and emotional status of working children
Miller 1997	Pakistan, India, Nepal, Bangladesh, and Sri Lanka	Boys and girls aged less than 15 years	Review of studies	Social class, gender, intrahousehold food allocation	Nutritional discrimination, female child mortality, sex ratios of surviving children, son preference, breastfeeding, malnutrition, stunting, and wasting
Gadit 1998	Urban-community mental health clinic, Karachi 1995-1997	200 boys and girls with severe depression	Clinical assessment, semi-structured interviews	Depression among abused children	Physical or sexual abuse, emotional deprivation, false implication in crimes, harassment by employer and social disadvantage
Mustansar 1998	Urban and rural Pakistan	Boys and girls aged less than 15 years	Review paper	Child labour	Reasons and types of child labour, and strategies to deal with this issue
Tahir 1998			Editorial	Child labour	Child labour terminology and interpretation
Talaat 1999	Urban Peshawar	230 working boys and girls aged 3-17 years (37.4% 15-17 years)	Simple questionnaire, observation	Child labour	Poverty, working conditions, lack of leisure and play, job security, malnutrition, and psychosocial problems
Channar 2000	Urban Bhawalpur	Boys and girls aged 7-15 years	Survey and interviews	Determinants of child labour	Socioeconomic conditions, poverty alleviation, compulsory education, work environment, ‘childhood’ as a right, and family planning
Mehnaz 2001	Urban Karachi	250 secondary school boys and girls	Survey, structured interviews, random sampling	Impact of street violence on children	Depression, sense of security, change in behavioural pattern, tendency towards aggression/ weapons for security
Aziz 2002	Pakistan	Boys and girls aged less than 18 years	Editorial	Violence against children	Child neglect, sex discrimination, and impact on health
Tabassum 2002	Squatter settlement/ peri-urban	150 working boys aged 12-14 years (mean=13.91 years)	Cross-sectional survey (systematic random sampling)	Child labour	Causes of child labour and occupational and health-related problems
Pakistan Pediatric Journal 2002	Pakistan	Boys and girls in the developing world	Editorial	Mental health services for children	Consequences of violence, psychiatric morbidity, and mental health services
Sethi 2002	Karachi city urban	112,029 child labourers aged 10 years or younger	Clinical screening	Child labour in Karachi	Reasons for working and working conditions

**Table 2:** Violence against children in Pakistan: reports from government, NGO, and international organizations

Organizational source	Setting	Characteristics of population studied	Methods	Primary topic	Secondary themes
UNICEF 1998	Pakistan, Bangladesh, India	Girls aged less than 18 years	Comparison of secondary data	Status of girl child	Education, health, income, rapes of minors, human traffcking, and sexual exploitation
Sahil 1998	Pakistan	Boys and girls aged less than 18 years	Review of print media reports	Child abuse	Identifcation and prevention of physical and sexual child abuse
Mehnaz A. Pakistan Pediatric Forum 2000	Pakistan	Boys and girls aged less than 18 years	Review of newspaper reports, hospital observations, and NGO data	Child abuse	Strategies to combat child abuse
Pakistan Pediatric Association Child's Right's Group 2002	Pakistan	Boys and girls aged less than 18 years	Review of data from organizations, print media, Chief Chemical Examiner's Office, and Police Surgeons Office	Child sexual abuse in Pakistan	Issues of collecting data, abuser categories, child development, and gender disparities
Society for the Protection of the Rights of the Child 2002	Pakistan	Boys and girls aged less than 18 years	Government and multilateral agencies’ reports and surveys	State of Pakistani children	Poverty, health, education, child labour, child rights, violence against children, birth registration, child sexual abuse, juvenile justice, and media violence
Human Rights Watch 2002	Global perspective	Boys and girls aged over 18 years	Review of ILO, UNICEF and World Bank reports	Children's rights	Developing countries, bonded child labour, labour laws, and child traffcking
Human Rights Commission of Pakistan 2003	Punjab province	Boys and girls aged less than 18 years	Reported cases	Child abuse	Sexual abuse, child abduction, and killings
UNICEF Pakistan 2003	Pakistan	Women and children	Review paper	The right's framework	Education, advocacy, health, and protection. Accountability and universality of the programmes
Raheela Asfa Undated	Pakistan	Boys and girls aged less than 18 years	Review of organizational reports	Role of UNICEF in preventing child abuse	Causes and possible prevention of child abuse and parental behaviour

ILO=International Labour Organization; NGOs=Non-governmental organizations; UNICEF=United Nations Children's Fund

Table 3 presents the understanding of violence against children as used in the literature in Pakistan. An important common feature to nearly all reviewed literature was their definition of violence against children, based on the United Nation's Convention on the Rights of the Child (CRC). In addition, the selected papers in Table 3 derived their own definition from CRC, which is either issue-specific or is an effort to apply the CRC to a type of violence against children. All definitions in the reviewed papers acknowledge four universal forms of abuse—physical, sexual, mental and neglect—resulting in actual or potential harm to health, survival, development, and dignity of the child, as constituting violence against children. The first two forms (physical and sexual) are elaborately defined and are deemed detectable because of their very obvious nature. However, neglect and psychological violence has been referred to as ‘suffering’, ‘intimidation by position of differential power’, ‘moral/ethical abuse’, and ‘harmful traditional practices’. In this context, the reviewed literature identified corporal punishment, impact of street violence on children, malnutrition, poverty, educational neglect, and abandonment, as different forms of neglect.

**Table 3:** Defnitions of violence against children used in Pakistan literature

United Nations Convention on the Rights of the Child (CRC)	
Defnition of a child: Child is recognized as a person under 18, unless national laws recognize the age of majority earlier. Non-discrimination: All rights apply to all children without exception. It is the State's obligation to protect children from any form of discrimination and to take positive action to promote their rights. Best interests of the child: All actions concerning the child shall take full account of his or her best interests. The State shall provide the child with adequate care when parents, or others charged with that responsibility, fail to do so. Implementation of rights: The State must do all it can to implement the rights contained in the Convention. Parental guidance and the child's evolving capacities: The State must respect the rights and responsibilities of parents and the extended family to provide guidance for the child which is appropriate to her or his evolving capacities. Survival and development: Every child has the inherent right to life, and the State has an obligation to ensure the child's survival and development.	
Organization/Article	Defnition
WHO World Report on Violence and Health 2002	“Child abuse or maltreatment constitutes all forms of physical and/or emotional ill-treatment, sexual abuse, neglect or negligent treatment or commercial or other exploitation, resulting in actual or potential harm to the child's health, survival, development or dignity in the context of a relationship responsibility, trust of power”
UNICEF	“Mistreatment, taking advantage of someone, using someone selfshly. As in making a child work to pay off their parent's debts or making them do dangerous or illegal work in order to make someone else better off. Child pornography and child prostitution are both examples of comercial sexual exploitation”
Society for the Protection of the Rights of the Child 2000, Pakistan	Violence against children encompasses all forms of physical and mental violence, injury or abuse, neglect or negligent treatment, harmful traditional practices, exploitation, bullying in schools, corporal punishment and sexual abuse. [Follow the Convention on the Rights of the Child]
Pakistan Pediatric Association Child Right's Group	There is no universal defnition of child abuse, and the concept varies from country to country and society to society. As a general guide, child abuse is defned as “any act of commission or omission that endangers or impairs a child's physical/psychological health and development. Such act is judged on the basis of a combination of community standards and professional expertise to be damaging. It is committed by individuals, singly or collectively, who by their characteristics (e.g. age, status, knowledge, organizational form) are in a position of differential power that renders a child vulnerable”
Mehnaz A *et al.* 2000	Defnition of violence in the context of law is ‘unlawful exercise of force’ and ‘intimidation by exhibition of force’
Aziz F 2002	“Child battering is not just physical abuse. It can be mental, emotional, sexual, moral and ethical abuse and perhaps in its most important and unrecognized subtle form, child neglect”
Gadit A 1998	Violence in terms of torture is defned as ‘deliberate, systematic infiction of physical or mental suffering by one or more persons acting alone or on the orders of any authority, to force another person to yield information, to make a confession or for any other reason”

UNICEF=United Nations Children's Fund; WHO=World Health Organization

A summary of all the suggested ‘reasons’ for child labour mentioned in 11 different studies relating to the issue in Pakistan are presented in Table 4. Some of these causes can be grouped together although no single category is mutually exclusive. For instance, family circumstances, unemployed parents, large family size, survival, and forced labour could be described as poverty-related causes. These working children seem to come from large, poverty-stricken families, with other factors, such as unemployment, drug addiction, migration, and illiteracy playing an important role. Another rationale proposed in the literature is weak legislation relating to both elementary education and child labour, including bonded labour. No quantitative estimates of the contribution of these causes, or estimates of risk were found in the studies. As a result, the commonality of their appearance has been used for illustrating the potential frequency of this cause.

**Table 4:** Suggested reasons/causes for child labour

Source	Family circumstances	Unemployed parents	Survival	Forced to work	Large family	Poverty	Weak child legislation	Inadequate elementary education	Bonded labour	Illiteracy	Rural to urban migration	Gender issues	Drug addict parents	Provide cheap labour	Work to earn skills	Own choice
Ahmed 1991	✓	✓	✓	✓	✓	✓	✓	✓		✓	✓			✓	✓	✓
Tabassum 2002	✓	✓	✓	✓	✓	✓	✓	✓	✓	✓	✓				✓	✓
Channar 2000	✓	✓		✓	✓	✓	✓	✓		✓		✓			✓	
Talaat 1996	✓	✓	✓	✓	✓	✓	✓	✓	✓	✓	✓	✓	✓	✓		✓
Talaat 1999	✓	✓	✓	✓	✓	✓	✓	✓	✓	✓	✓		✓			✓
Sethi 2002	✓	✓			✓	✓										
Mustansar 1998					✓	✓	✓	✓	✓	✓	✓					
Chhabra 1998						✓		✓		✓		✓				
Human Rights Watch																
2002	✓			✓		✓			✓							
SPARC 2002	✓	✓		✓	✓	✓	✓	✓	✓	✓	✓			✓		
UNICEF 2003	✓	✓	✓		✓	✓	✓	✓		✓	✓					

SRARC=Society for the Protection of the Rights of the Child; UNICEF=United Nations Children's Fund

Quantitative data were available from six studies only on child labour (Table 5). These papers suggest that children start work as early as three years of age, while the median age of entering the workforce was reported to be seven years by two studies (14,15). Certain types of work were common across studies, such as domestic employment (household labour), work in mechanic shops, small hotels or restaurants, and work in fruit markets with the youngest children reported to be part of ‘beggar’ groups. Children work for long (6–15) hours without breaks or leisure time, and sometimes children sleep at their work place in highly inadequate living conditions. Children work for very low wages, sometimes only for food in return, with their contribution to monthly household income ranging from 1% to 29%. The majority of these children are either completely illiterate or poorly educated. 10-60% of children reported physical abuse at the work place, while two studies reported that 66-79% of children felt pushed into work.

**Table 5:** Violence against children in Pakistan: quantitative estimates∗

Source	Children abused (rate per 100,000)	Raped/ sodomy %	Murdered %	Gang raped/sodomized %	Seriously injured %	Other %
Sahil 2003	2.62	25.72	7.83 (murdered after some form of sexual abuse)	20.08		34.45 (abducted) 11.92 (molested)
Sahil 1998	1.57		9.30	30		
Pakistan Pediatric Association 1999	1.00	100				
SPARC 2002	3	21	35	18	16	
Human Right's Commission of Pakistan 2003	1.26	52	23 (4.7% sexual assault)	<1		26 (abducted)
Madadgaar 2003	3.18	27 (including attempts)	34	15	14	3 (tortured)

∗Rates have been adjusted for annual reporting periods and are based on a reference population of Pakistan aged less than 15 years); SPARC=Society for the Protection of the Rights of the Child

We have quantitative data from five organizations on physical and sexual child abuse from 1998 to 2003 (Table 5). Three organizations reported national data, and two focused on statistics from Punjab province and Lahore city. However, these organizations made it clear that these data did not represent actual numbers of such incidents in Pakistan because of underreporting. Reasons, such as family honour, concepts of morality, and cultural taboos, were major reasons for underreporting. The three national reports indicated a higher proportion of boys in those reportedly abused, while the two reports from Punjab reported a higher proportion of girls. Using a reference national population (children aged less than 15 years) as of 1998 (latest census available) for the area of study, rates of annual incidence have been generated. These rates assume uniform reporting across the year and have not been adjusted for underreporting to derive ‘minimalist’ estimates. The reported annual incidence of violence against children ranged from 1.57 to 3.18 per 100,000 for Pakistan. The categories—physical and sexual abuses—used in these reports included abduction, rape, sodomy, torture, or murder against both boys and girls. As can be seen, rape and murder turned out to be most frequent causes, while fewer proportions appeared in the abduction and torture category (Table 5). The category of ‘seriously injured’ is mentioned but not defined in the studies.

## DISCUSSION

Violence against children needs to become a public-health priority worldwide. This review showed that there is some, though limited literature (published or unpublished) on violence against children in Pakistan. The literature is mostly focused on the causes and, in some cases, the consequences of child abuse. Most studies and reports have documented child labour, child sexual abuse, and fatal violence, whereas neglect or other non-physical forms of violence have not been captured. The reviewed papers tended to focus on urban settings in the country, while the majority (70%) of the population of Pakistan lives in rural areas. Most work was of observational and descriptive nature with data collected through survey methods and interviews with small sample sizes. The time periods of reporting also vary, and methodological details are often not available in the papers. The duration of reporting differs in all cases from seven months to (over a) one year period. The method of collecting such cases also differed from police reports to special surveillance of newspaper-reported cases. The findings reflect the need for more and better-quality information on violence against children in Pakistan.

All studies in this review present definitions of violence against children that are derived from the United Nations Convention on the Rights of the Child (18). This common understanding is very welcome and is important because it allows a universal approach with flexibility to address sociocultural contexts. Although child abuse per se can be defined universally, the diverse types of abuse, their existence in different societies, and more importantly interventions for its prevention and control need to focus on the specific cultural contexts within each country. Pakistan presents a specific set of evolving conservative and traditional social structures, which are reflected in the literature. This review identifies that children in Pakistan experience numerous risk factors in their exposure to the outside world and the quality of care provided by their immediate families. Cultural and traditional norms that foster gender discrimination and under-value the girl child contribute to high rates of overall illiteracy, low nutritional status, and lack of access to health and development opportunities in life (29). This represents a hostile macro context, which leads to an increased risk of violence against children (rape, human trafficking, and sexual exploitation). Corporal punishment and the slow process of justice for children who come in contact with the law are another form of neglect at the institutional and societal levels. Dimensions of household neglect, such as improper supervision, abandonment, and educational deprivation, have not been elaborated in the reviewed papers.

The literature review also explored the causes of child labour and its impact on children, their families, and overall human development. There is a strong qualitative association between poverty and related factors (family size, unemployment) with child labour in Pakistan. Studies on child labour indicate that, for these children, living in the cycle of a poor large family with unemployed parents creates the conditions in which they have to work. Illiteracy, lack of educational opportunities, and weak legal structures then further disadvantage these children, making them the lowest paid workers in the country. Working children then experience harsh working conditions, lack of food and rest, and no play which then disproportionately affect their health. Studies documented children reporting complaints of skin and eye infections, problems relating to the digestive system, headaches, dizziness, asthma, body aches, stress, and depression. These health outcomes were not specifically diagnosed in the studies nor were the presence of other health consequences, such as suicide, studied. In addition to putting children in physical danger, work reduces their chances of getting an education. An early-age exposure to ‘street survival situations’ also makes them at risk for drug abuse, prostitution, and crime.

Studies that provided quantitative data on physical and sexual abuses of children are challenging to interpret. Studies relied on cases of abuse that were either reported in newspapers or also reviewed police records (16,22). Newspapers have been used as sources of health data in Pakistan and for reporting stigmatized events more frequently than the police (29). The use of such data in deriving a rate of child abuse is only meant to capture a ‘minimalist’ estimate of the problem and to stimulate a research agendum for child health in Pakistan. It was not clear whether the trend in available ‘estimates’ of child abuse, from 1998 to 2003, was because of more events happening or simply because of better reporting. This distribution of reported cases by gender provided an inconsistent pattern across the studies reviewed here. What is clear is that both girls and boys are victims in Pakistan. The usual reason given for not reporting sex-related crimes is that it has a huge social stigma attached to it, not only for the victim but also for the victim's family. Talking about sex in the conservative Pakistani society is taboo; nevertheless, sexual violation and exploitation of children is happening. It is important that this problem is not just acknowledged, but also explored in terms of its magnitude and impact, and national-level data inform policies and strategies for prevention and control.

The concept of post-event care and victim support for child victims barely exists in Pakistan. Non-governmental organizations have initiated limited support services in some urban parts of the country but there are no data to determine either their accessibility or their effectiveness. Moreover, there are no national centres or publicly-subsidized services of this type across the nation. Our review indicates that weak legislation on elementary education, poor labour laws, and corporal punishment are also a determinant of an increasingly complex situation within which violence against children is perpetrated. This goes beyond health, economics, and development to become a political question; a continued lack of political will is, thus, only going to distance the children of Pakistan from realizing their potential.

The confluence of macro risk factors, such as poverty, poor legal protections, and illiteracy, together with family specific factors, such as large size and unemployment, create an enabling environment for violence against children. Focused research is needed to examine the prevalence, manifestations, and potential interventions for violence against children from a public-health perspective. The health problems reported by children and the extent of human potential destroyed are unknown. It is imperative that healthcare providers find alternative ways to identify and address violence as an issue threatening the future of children. It is crucial for policy-makers in Pakistan to recognize that children are particularly vulnerable to violence and that ignoring child rights only further threatens their health and development while they are trapped in a cycle of poverty and helplessness.
